# COVID-19 Inflammatory Syndrome: Lessons from TNFRI and CRP about the Risk of Death in Severe Disease

**DOI:** 10.3390/biomedicines12092138

**Published:** 2024-09-20

**Authors:** Thaís Soares Farnesi-de-Assunção, Ana Carolina de Morais Oliveira-Scussel, Wellington Francisco Rodrigues, Beatriz Sodré Matos, Djalma Alexandre Alves da Silva, Leonardo Eurípedes de Andrade e Silva, Fabiano Vilela Mundim, Fernanda Rodrigues Helmo, Anna Victória Bernardes e Borges, Chamberttan Souza Desidério, Rafael Obata Trevisan, Malu Mateus Santos Obata, Laís Milagres Barbosa, Marcela Rezende Lemes, Juliana Cristina Costa-Madeira, Rafaela Miranda Barbosa, Andrezza Cristina Cancian Hortolani Cunha, Loren Queli Pereira, Sarah Cristina Sato Vaz Tanaka, Fernanda Bernadelli de Vito, Ivan Borges Monteiro, Yulsef Moura Ferreira, Guilherme Henrique Machado, Hélio Moraes-Souza, Denise Bertulucci Rocha Rodrigues, Carlo José Freire de Oliveira, Marcos Vinicius da Silva, Virmondes Rodrigues Júnior

**Affiliations:** 1 Department of Microbiology, Immunology and Parasitology, Federal University of Triângulo Mineiro, Uberaba 38025-180, MG, Brazil; thais.assuncao@uftm.edu.br (T.S.F.-d.-A.); ana.morais@uftm.edu.br (A.C.d.M.O.-S.); wellington.frodrigues60001@gmail.com (W.F.R.); beatrizsodre123@gmail.com (B.S.M.); djalma.bio2@gmail.com (D.A.A.d.S.); fabianouftm@gmail.com (F.V.M.); fernanahelmo@gmail.com (F.R.H.); annab.borges@outlook.com (A.V.B.e.B.); chamberttan_sd@hotmail.com (C.S.D.); rafael_obata1@hotmail.com (R.O.T.); malumateus26@gmail.com (M.M.S.O.); laism.bio@gmail.com (L.M.B.); marcela.lemes.r@gmail.com (M.R.L.); julianamadeiravet@gmail.com (J.C.C.-M.); rafaelamirandabarbosa98@gmail.com (R.M.B.); carlo.oliveira@uftm.edu.br (C.J.F.d.O.); marcos.silva@uftm.edu.br (M.V.d.S.); 2 Clinical Analysis and Pathological Anatomy Laboratory, Empresa Brasileira de Serviços Hospitalares (EBSERH), Federal University of Triângulo Mineiro, Uberaba 38025-180, MG, Brazil; leonardoeuripedes@gmail.com; 3 Hematological Research Laboratory, Federal University of Triângulo Mineiro, Uberaba 38025-180, MG, Brazil; andrezzaccunha@gmail.com (A.C.C.H.C.); lorenbiomedica@gmail.com (L.Q.P.); sarah.tanaka@uftm.edu.br (S.C.S.V.T.); fernanda.vito@uftm.edu.br (F.B.d.V.); helio.moraes@uftm.edu.br (H.M.-S.); 4 UNIMED São Domingos Hospital, Uberaba 38025-110, MG, Brazil; ibmonteiro05@yahoo.com.br; 5 Alencar Gomes da Silva Regional Hospital, Uberaba 38060-200, MG, Brazil; yulsef@gmail.com; 6 Mário Palmério University Hospital, Uberaba 38050-501, MG, Brazil; guilhermehm7@hotmail.com; 7 Centro de Formação Especial em Saúde (CEFORES), Federal University of Triângulo Mineiro, Uberaba 38025-180, MG, Brazil; denise.rodrigues@uftm.edu.br; 8 Department of Immunology, Medical School, University of Uberaba, Uberaba 38010-200, MG, Brazil

**Keywords:** COVID-19, *SARS-CoV-2*, clinical outcomes predictor, inflammatory mediators, TNF superfamily, TNF receptors, C-reactive protein

## Abstract

**Background/Objectives**: Cytokine storm in severe COVID-19 is responsible for irreversible tissue damage and death. Soluble mediators from the TNF superfamily, their correlation with clinical outcome, and the use of TNF receptors as a potent predictor for clinical outcome were evaluated. **Methods**: Severe COVID-19 patients had the levels of soluble mediators from the TNF superfamily quantified and categorized according to the clinical outcome (death versus survival). Statistical modeling was performed to predict clinical outcomes. **Results**: COVID-19 patients have elevated serum levels from the TNF superfamily. Regardless of sex and age, the sTNFRI levels were observed to be significantly higher in deceased patients from the first weeks following the onset of symptoms. We analyzed hematological parameters and inflammatory markers, and there was a difference between the groups for the following factors: erythrocytes, hemoglobin, hematocrit, leukocytes, neutrophils, band cells, lymphocytes, monocytes, CRP, IL-8, IFN-γ, IL-10, IL-6, IL-4, IL-2, leptin MIF sCD40L, and sTNFRI (*p* < 0.05). A post hoc analysis showed an inferential capacity over 70% for some hematological markers, CRP, and inflammatory mediators in deceased patients. sTNFRI was strongly associated with death, and the sTNFRI/sTNFRII ratio differed between outcomes (*p* < 0.001; power above 90%), highlighting the impact of these proteins on clinical results. The final logistic model, including sTNFRI/sTNFRII and CRP, indicated high sensitivity, specificity, accuracy, and an eight-fold higher odds ratio for an unfavorable outcome. **Conclusions**: The joint use of the sTNFRI/sTNFRII ratio with CRP proves to be a promising tool to assist in the clinical management of patients hospitalized for COVID-19.

## 1. Introduction

*SARS-CoV-2* infection, which has been responsible for millions of deaths, continues to be the focus of recent research aimed at filling gaps in our understanding. Numerous variables influence the severity and progression of COVID-19 [1]. Among these, the host’s immune response plays a crucial role in the disease’s pathogenesis, since an excessive and uncontrolled production of cytokines can lead to irreversible damage, multiple organ failure, and, ultimately, death [2,3]. The presence of a cytokine storm has been associated with the severity of COVID-19, as severe cases often exhibit elevated concentrations of various cytokines [3,4].

The cytokine storm mediated by viruses increases various pro-inflammatory molecules. Individuals affected by *H5N1* exhibit abnormal levels of IL-8, IP-10, MCP-1, MIP-1, MIG, and CXCL9, while those with severe MERS (Middle East Respiratoty Syndrome) show significantly elevated serum levels of IL-6, IFN-γ, TNF-α, IL-15, IL-17, IL-8, CXCL10, and CCL15 [3]. The dysregulated production of inflammatory mediators is associated with organ dysfunction in COVID-19 [4].

Among the cytokines, TNF superfamily members stand out, such as the key proteins involved in the cytokine storm, and are inherently pro-inflammatory molecules with significant roles in viral infection processes [5]. TNF-α, a molecule closely associated with inflammation, can trigger PANoptosis while working in synergy with IFN-γ [6]. Furthermore, it is associated with pulmonary edema in COVID-19, as it mediates the breakdown of epithelial barrier integrity [7]. The effects of TNF-α are initiated by its interaction with its receptors, TNFRI and TNFRII, which display a differential expression in various tissues and cells. These receptors possess extracellular domains, which are similar to each other and rich in cysteines, as well as characteristic intracellular domains, all of which contribute to their distinct and specific biological functions [8,9].

Despite a decline in the number of COVID-19 cases and in the mortality rate, severe infections continue to pose a risk to the lives of affected individuals. Therefore, identifying effective biological markers that can predict disease severity is imperative for more precise therapeutic interventions as well as the development of biological tests and predictive scores that can significantly improve clinical management. Some inflammatory mediators, when adjusted for clinical, demographic, and laboratory factors, have demonstrated good prognostic capabilities [10]. However, until now, there is limited research that has provided insights into how often correct predictions occur in the studied population. Therefore, in this paper, we analyzed soluble inflammatory mediators from the TNF superfamily to assess if they can directly influence clinical decision making when used as predictors for the clinical outcomes of COVID-19 patients in combination with routine laboratory tests and statistical modeling.

## 2. Materials and Methods

### 2.1. Design Study and Participants

Individuals who required hospital care due to COVID-19 were invited to participate in this study. These individuals were admitted to reference hospitals for the treatment of COVID-19 in Uberaba, Minas Gerais State, Brazil, in the year 2020. The diagnosis of the infection was based on a positive result for *SARS-CoV-2* by RT-PCR, clinical signs and symptoms, chest computed tomography findings, and clinical expert consensus. Initially, these patients were categorized into two groups: patients with active disease (Active Patients group: AP/COVID-19^pos^). Further subdivisions were made within the Active Patients group, including Survivor Patients (Survivor Patients group: SP/COVID-19^pos^) and Deceased Patients (Deceased COVID-19 Patients group: DP/COVID-19^pos^). Additionally, healthy individuals without a prior history of *SARS-CoV-2* infection or immunological diseases were also recruited for this study (Healthy Donor group: HD/COVID-19^neg^).

This study was conducted in compliance with the Declaration of Helsinki and was approved by the National Research Ethics Commission (CAAE No. 30474020.2.0000.0008). All participants (or their legal representatives) provided their informed consent by signing a free and informed consent form. Individuals who did not sign the consent form were excluded from the study.

### 2.2. Procedures and Cytokine Measurement Methods

Serum samples were collected from hospitalized patients during their hospitalization. The blood was drawn into tubes without anticoagulant, followed by centrifugation, and the resulting serum was stored at −80 °C until it was ready for quantifying soluble factors. Clinical data about the hospitalized patients were obtained from their medical records, and additional information was gathered through personal communication with healthy donors.

The concentrations of soluble factors were measured with the help of enzyme-linked immunosorbent assay (ELISA) and cytometric bead array (CBA). The data were collected using an absorbance reader, EnSpire^®^ (PerkinElmer, Cambridge, MA, USA), or a flow cytometer, FACS Calibur, before being analyzed on FCAP Array 2.0 software (BD Biosciences, San Jose, CA, USA). In both cases, the concentrations of cytokines and soluble factors were determined based on a standard curve. For more specific details regarding the measurement methods, please refer to the Supplementary Materials (Table S1).

### 2.3. Statistical Analysis

The tests used are described in the legend for each table and figure presented in this study. Analysis was performed using GraphPad Prism 8.0 Software (GraphPad Software, San Diego, CA, USA). G*Power version 3.1.7 (Uiversität Kiel, Kiel, Germany) was also used for sampling estimates and power of inferences (post hoc). The data were tabulated in a Microsoft^®^ Excel (Microsoft Office Professional Plus 2016, Redmond, WA, USA) program and analyzed using IBM SPSS Statistics 27 (IBM Corp., Armonk, NY, USA). The evaluation of the distribution was performed using the Shapiro–Wilk test, whereas the homoscedasticity was assessed through Levene’s test. Welch correction was used for cases of unequal variances.

To generate association indicators, multivariate evaluations were initially conducted. MANOVA was applied to verify possible significant associations between the study parameters and disease outcome. The multivariate tests Pillai’s trace, Wilks’ lambda, Hotelling’s trace, and Roy’s largest root were used. Additionally, principal component analysis and classification tree (growing method: CHAID) were used to evaluate potential clusters and relationships to the outcome.

Fisher’s exact test was employed to analyze differences in a 2 × 2 contingency table, such as the sex of study groups and subgroups. The distribution was evaluated using the Shapiro–Wilk test. Comparisons between groups (female versus male and survivor versus deceased) were conducted with an unpaired t-test (for parametric data) or Mann–Whitney test (for non-parametric data). The ANOVA or Kruskal–Wallis test with Dunn’s post-test was conducted for a comparison between groups greater than three (e.g., sex of survivor and deceased), for parametric and non-parametric data, respectively. The multiple t-test was used to analyze differences in soluble factor kinetics between weeks of infection evolution. Mann–Whitney U test or the unpaired t-test was used to compare outcomes (discharge or death). Furthermore, the rank biserial correlation or Cohen’s d was used to determine the sizes of the effect. Multivariate logistic regression analyses were conducted to initially verify statistically significant associations between the variables and the outcome. Subsequently, a binomial logistic regression model was used to predict and estimate the effects (odds ratios [ORs] and confidence intervals [CIs]) of different potential explanatory variables for the death outcome. The parameters selected for modeling fulfilled the following three criteria: significant differences between potential explanatory variables, power of statistical inferences of at least 70% (refers to their ability to detect genuine effects or differences, based on the sampling design and statistical test applied), and tolerance of 80%. The Akaike information criterion (AIC), Schwarz Bayesian criterion (BIC), and R^2^ of McKelvey were also utilized to assess the complexity and adherence of the models. The accuracy, specificity, sensitivity, and AUC were determined for the predictive model presented [11,12,13,14,15]. A significance level of 5% was considered in all analyses. Lastly, the experimental design, database, methods, and data analysis were confirmed by a statistician.

## 3. Results

### 3.1. Study Population

Hospitalized patients, in 2020, with active *SARS-CoV-2* infection (*n* = 214) were included in this study (mean age: 58.42 ± 16.07 years, Table S2). This group includes individuals who suffered from moderate and severe COVID-19 and thus required hospital care, whether for ventilatory support and/or highly complex medical care. After analyzing the data comparing healthy donors (HD/COVID-19^neg^) and those with active disease (AP/COVID-19^pos^), differences in terms of sex are evident (*p* = 0.0387, Table S2). Among them, 124 patients survived (SP/COVID-19^pos^ group) and 90 were deceased (DP/COVID-19^pos^ group), with a mean age of 53.44 ± 14.21 years and 65.14 ± 16 years, respectively (*p* < 0.0001, Table S2). The analysis of demographic data revealed no difference between the sexes; however, age proved to be an exception, which, regardless of sex, was found to be higher in individuals who passed on (*p* = 0.0414 for females and 0.0006 for males, Table S2). The patients in this study had provided at least one venous blood sample. The time, in days, between the onset of symptoms and the first blood collection was similar in both cohorts, with an average of 8.0 and 9.0 days for the DP/COVID-19^pos^ and SP/COVID-19^pos^ groups, respectively (Table S2). Furthermore, the average time, in days, between the first symptoms and the clinical outcome revealed no statistical difference between the groups. The DP/COVID-19^pos^ group showed an average of 19.0 days between the onset of symptoms until the outcome, while the SP/COVID-19^pos^ group depicted an average of 15.0 days (Table S2).

We evaluated the blood count data of the 214 patients included in this study, corresponding to the date on which the first blood sample was collected. For the red blood cell series, the DP/COVID-19^pos^ group showed decreased red blood cells (*p* = 0.0048), hemoglobin percentage (*p* = 0.0053), and hematocrit percentage (*p* = 0.0183) when compared to the SP/COVID-19^pos^ group (Table S3). In the white blood cell series, the DP/COVID-19^pos^ group presented lymphopenia (*p* < 0.0001) and showed an increase in polymorphonuclear (*p* = 0.0093) compared to the SP/COVID-19^pos^ group (Table S3). Furthermore, lymphopenia was more pronounced in men impacted by COVID-19 (*p* = 0.0243, AP/COVID-19^pos^ group), with a significant reduction in the DP/COVID-19^pos^ group (*p* = 0.0056) compared to females (Table S3). The DP/COVID-19^pos^ group also depicted a reduction in monocytes compared to the SP/COVID-19^pos^ group (*p* = 0.0071, Table S3). No changes were observed in total leukocytes and platelet series concerning outcome and sex (Table S3).

Initial multivariate analyses revealed a significant association for the selected biomarkers in the study (Pillai’s trace = 0.82, *p* = 0.002; Wilks’ lambda = 1.69, *p* < 0.001; Hotelling’s trace = 1.35, *p* < 0.001; and Roy’s largest root = 0.95, *p* < 0.001). The model used for the classification tree linked to inflammatory biomarkers indicated three clusters with significant power for separating the outcome of death or hospital discharge related to TNFRI.

### 3.2. TNF Family Soluble Factors

The study included multiple venous blood collections from COVID-19 patients during their hospitalization period. To minimize the potential interference of time and hospital stay length on the immune system activation, the analysis primarily focused on the data obtained from the first venous blood collection of each patient.

**Figure 1:**
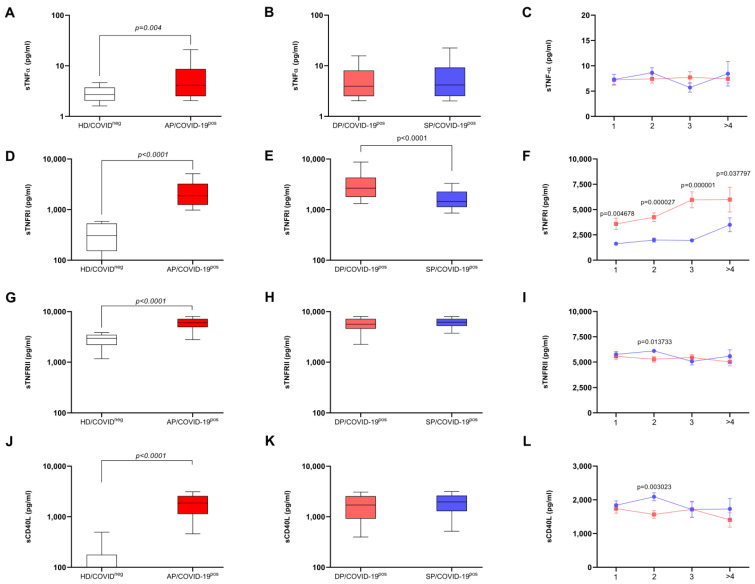
TNF family soluble factors, their relationship to COVID-19 active patient and their kinetic in weeks, after symptoms begin, according to clinical outcomes. TNF family soluble factor levels in the serum of healthy individuals and patients with active COVID-19 by the ELISA method. The results are expressed in pg/mL. In (**A**,**D**,**G**,**J**), soluble TNF-α, TNFRI, TNFRII, and CD40L levels can be seen, respectively, according to the clinical diagnosis (healthy and active COVID-19). In (**B**,**E**,**H**,**K**), soluble TNF-α, TNFRI, TNFRII, and CD40L levels are observed, respectively, according to the clinical outcomes (death and discharge). Patients with active disease AP/COVID-19^pos^ group, *n* = 214), patients with active disease and evolution to death (DP/COVID-19^pos^ group, *n* = 90), patients with active disease and evolution to hospital discharge (SP/COVID-19^pos^ group, *n* = 124), and healthy donors (HD/COVID-19^neg^ group, *n* = 14). The horizontal line represents the median; the bar shows the 25% to 75% quartiles, whereas the vertical line depicts the 10% and 90% percentiles. The statistical differences between groups are expressed in the graphic. In the graphs on the right, the *p*-values above the boxplot refer to the statistical differences in relation to the control group (healthy donors). Mann–Whitney test, Kruskal–Wallis test, Dunn’s post-test, and ANOVA were used for this study. In (**C**,**F**,**I**,**L**), soluble TNF-α, TNFRI, TNFRII, and CD40L levels are shown, respectively, following the symptom onset in weeks, according to the clinical course (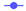 survivor and 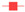 death). Patients with active disease and evolution to death (DP/COVID-19^pos^ group, *n* = 168) and patients with active disease and who survived (SP/COVID-19^pos^ group, *n* = 154) are also presented. The points represent standard error of the mean (SEM). The statistical differences between groups during the weeks are expressed in the graphic. Multiple t-tests were used, and the results were considered statistically significant at *p* < 0.05.

The initial analysis revealed that *SARS-CoV-2* infection significantly increased serum levels of TNF family soluble factors, specifically sTNFα, sTNFRI, sTNFRII, and sCD40L when compared to healthy individuals’ serum levels (HD/COVID-19^neg^ group) (*p*-values: sTNFα = 0.004, sTNFRI < 0.0001, sTNFRII < 0.0001, sCD40L < 0.0001, Figure 1A, Figure 1D, Figure 1G and Figure 1J, respectively).

When the COVID-19 patients were further categorized based on clinical outcomes (survivor or deceased), it was evident that both survivor and deceased groups depicted a significant serum increase in these mediators compared to the healthy group. However, the sTNFRI soluble form was significantly elevated in the group with an unfavorable clinical outcome (deceased patients) when compared to the survivor group (*p* < 0.0001, Figure 1E).

The study also explored the influence of age and sex on the immunological mediators’ serum levels (Tables S4–S7). The findings indicate that sTNFRI levels were significantly higher in both males and females who died due to *SARS-CoV-2* infection (*p* < 0.001, Table S5). Additionally, elderly individuals over the age of 60 were found to have higher levels of sTNFRI (Table S6). Notably, sTNFRI levels were consistently elevated in deceased individuals, regardless of their age (below 59 years or above 60 years, Table S7).

Under the analyzed conditions, the levels of sTNF, sTNFRII, and sCD40L did not show significant differences between the groups (Tables S4–S7), except for decreased serum levels of sCD40L in deceased individuals over 60 years of age (*p* = 0.0229, Table S7).

This information provides valuable insights into the relationship between the immune response and clinical outcomes in COVID-19 patients, with a focus on age and sex as potential contributing factors.

The analysis of changes in serum levels over the weeks of hospitalization included all samples from patients in the study, amounting to 322 specimens. Of these, 168 samples were from the DP/COVID-19^pos^ group (deceased patients), and 154 samples were from the SP/COVID-19^pos^ group (survivor patients). The samples were grouped based on the number of weeks following the onset of symptoms.

The cytokine kinetics revealed that individuals who ultimately died had elevated sTNFRI levels from the first week after the onset of symptoms, and this elevation was maintained over subsequent weeks (Figure 1F). The statistical analysis demonstrated significant differences in sTNFRI levels at various time points after the onset of symptoms: first week: *p* = 0.004678; second week: *p* = 0.000027; third week: *p* = 0.000001; and fourth week: *p* = 0.0037797 (Figure 1F). These data indicate a sustained increase in sTNFRI levels in individuals who eventually did not survive the COVID-19 infection, starting as early as the first week after the onset of symptoms.

### 3.3. Effects of Biomarkers among COVID-19 Patients

To assess the impact of biomarkers on patients who either survived or succumbed to severe COVID-19, data from 322 samples were analyzed, including blood counts, C-reactive protein (CRP) (Table 1), and various inflammatory molecules (Table 2 and Table 3). In the group of patients who did not survive, significant differences were observed in several blood count parameters, including red blood cells (*p* < 0.001), hemoglobin % (*p* < 0.001), hematocrit percentage (*p* < 0.001), leukocytes (*p* < 0.001), neutrophils (*p* < 0.001), band cells (*p* = 0.031), lymphocytes (*p* < 0.001), and CRP (*p* < 0.001), compared to survivors. These variables demonstrate an inference power above 70%, emphasizing their substantial impact (Table 1).

Furthermore, various soluble inflammatory mediators were notably elevated in patients who did not survive, including IL-8 (*p* = 0.032), IL-10 (*p* = 0.002), IL-6 (*p* < 0.001), and MIF (*p* < 0.001, Table 2). Additionally, sCD40L was lower in patients who succumbed to the disease (Table S7 and Table 2). Other soluble mediators such as IFN-γ (*p* = 0.007), IL-4 (*p* = 0.002), IL-2 (*p* = 0.048), and leptin (*p* = 0.011) were also present in higher levels in this group (Table 2). Despite these findings, most variables showed an inferential power of less than 62% (Table 2).

Soluble TNFRI was significantly associated with death in COVID-19 patients (*p* < 0.001), and the ratios TNFRI/TNFα and TNFRI/TNFRII also depicted significant differences between the two groups (*p* < 0.001, Table 3). The effect size of these associations was verified, with an inference power greater than 90%, indicating a robust impact of these parameters on the outcome (Table 3).

### 3.4. Soluble TNFRs Predict Prognosis of Patients with Severe COVID-19

In the search for prognostic indicators in COVID-19 patients, logistic regression models were initially applied using the study variables. Although some variables showed differences between subgroups in inferential analyses, most did not demonstrate sufficient discriminative power between outcomes when grouped. Therefore, only variables with predictive potential were considered and are presented here. Variables such as hemoglobin, neutrophils, CRP, and sTNFRI were selected for modeling due to their predictive relevance (Table S8). However, after collinearity analysis, it was observed that both TNFRI and the TNFRI/TNFRII ratio held similar strengths in the model, leading to the exclusion of TNFRI. Therefore, a new model was constructed using the TNFRI/TNFRII ratio (Table 4).

The resulting model demonstrated that for every 10 mg/dL increase beyond the CRP reference value, the risk of death in COVID-19 patients increased by 7.3% (OR = 1.0073; CI = 1.002 to 1.01; *p* = 0.011). Simultaneously, the TNFRI/TNFRII ratio demonstrated a significant association with a 7.2332 times higher chance of death (OR = 7.2332; CI = 1.857 to 28.17; *p* = 0.004). This model achieved an 83% correct probability estimate (AUC = 0.83), with 74% accuracy, 87% specificity, and 52% sensitivity (Table 4).

In a refined model excluding a few variables (hemoglobin and neutrophils), the AUC remained high at 0.81, indicating an 81% correct probability estimate. This model showed improved accuracy (77%), specificity (90%), and sensitivity (59%) compared to the previous model. The CRP analysis in this model revealed that for every 10 mg/dL increase beyond the reference value, the risk of death increased by 8.4% (OR: 1.0084; CI = 1.0032 to 1.014; *p* = 0.002). Additionally, the TNFRI/TNFRII ratio indicated an approximately 8.3 times higher risk of death in COVID-19 patients (OR = 8.3405; CI = 2.4201 to 28.745; *p* < 0.001) when both variables were considered together (Table 4).

**Figure 2:**
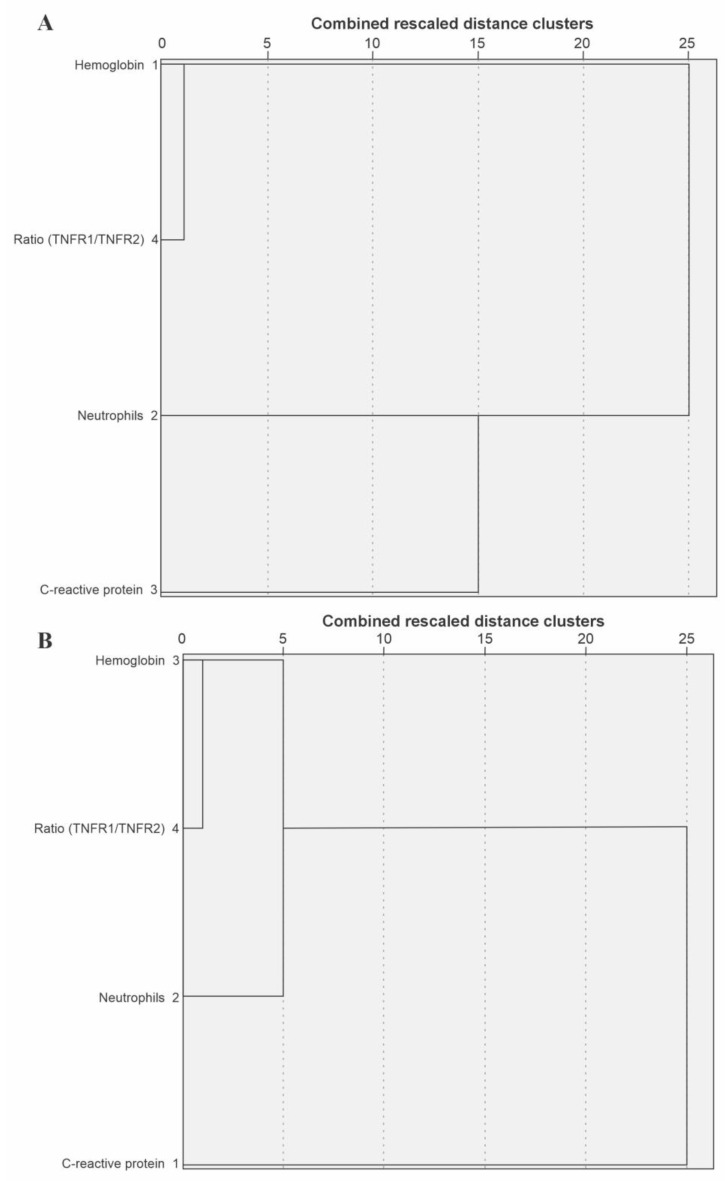
Graphical representation for the dis(similarity) matrix between the explanatory variables for the association with the prognosis of symptomatic patients for COVID-19. A dis(similarity) matrix was generated for the explanatory variables using a multivariate grouping method, the Euclidean distance. In (**A**), the distribution of groups for patients whose outcome was discharge. In (**B**), matrix data for the groupings of patients whose outcome was death. TNFRI: tumor necrosis factor receptor I; TNFRII: tumor necrosis factor receptor II; CRP: C-reactive protein.

A separate model constructed solely using the TNFRI/TNFRII ratio revealed an odds ratio of 3.974 for an unfavorable outcome in a COVID-19 patient (OR = 3.974; CI = 2.070 to 7.628, *p* < 0.001). Despite slightly lower values for accuracy (0.68) and sensitivity (0.59), this model showcased higher specificity (0.92, Table 4). The dis(similarity) between the explanatory variables can be observed in the dendrogram (Figure 2).

## 4. Discussion

The study initially focused on evaluating various serum soluble factors from healthy individuals and the TNF superfamily in COVID-19 patients requiring hospital care. The analysis included inflammatory kinetics concerning clinical outcomes during weeks of hospitalization, effect size assessments of these molecules, and statistical modeling to predict mortality.

Demographic findings reveal a higher prevalence of males among COVID-19 patients, and individuals over 60 years old were more likely to succumb to the disease. Age, especially over 65, correlated with a threefold increase in the risk of death [16,17]. Patients who died exhibited changes in hematological indicators, such as reductions in the red series, lymphopenia, monocytopenia, and neutrophilia. Thrombocytopenia and thrombotic events were not identified in the platelet series; however, they are prevalent in severe COVID-19 [18,19,20].

The study delved into biomolecules involved in host defense, specifically the TNF superfamily cytokines. Regardless of the clinical outcome, hospitalized COVID-19 patients depicted elevated serum concentrations of sTNF-α, sTNFRI, sTNFRII, and sCD40L. Previous studies have associated elevated sTNF-α and sTNFR levels with COVID-19 [21,22,23]. Others associated sTNF-α levels with pulmonary COVID-19 [7]. A study also highlighted the role of ADAM17, a protease involved in the release of these soluble factors into circulation, and its increased activity in severe COVID-19 cases [23].

These findings contribute to the understanding of the pathophysiology of COVID-19, emphasizing the importance of inflammatory mediators and shedding light on potential prognostic indicators for severe outcomes.

Patients with severe COVID-19 require intensive care and are more likely to use invasive ventilatory support and succumb to the illness. Therefore, the cohort was analyzed in relation to clinical outcome (survivors vs. deceased). Previously, elevated levels of TNFR1, TNFR2, and CD40L have been associated with patients admitted to intensive care units and the use of invasive mechanical ventilation [21,22,24], in addition to being correlated with the severity of the disease, when scaled into mild, moderate, and severe cases [21]. From this perspective, the study demonstrates that regardless of sex and age, the deceased patients had even higher sTNFRI levels compared to those with a favorable outcome, in addition to being more evident in the first weeks after the onset of symptoms.

TNFRI is constitutively expressed in nucleated cells, whereas TNFRII is restricted to hematopoietic and endothelial cells [5,25]. Both receptors are activated by TNF, with TNFRI predominantly activated by the soluble form [5,26]. TNFRI, known as a death receptor [8], triggers pro-inflammatory cytokine production upon binding with sTNFα and subsequently leads to cell apoptosis or necroptosis [27,28]. This process contributes to lymphopenia observed in severe COVID-19 and activates the inflammasome [29,30], leading to increased IL-1β levels and neutrophilia.

The study highlights the intricate balance of TNF and its receptors in COVID-19 pathogenesis. Although controlled and local TNF/TNFRI activation aids infection resolution and tissue repair, excess TNF can be harmful [26]. The soluble forms of TNF and its receptors, particularly sTNFRI, act as a protective mechanism against TNF excess [5]. However, the complex formed by sTNF-α/sTNFR may act as a reservoir, influencing the long-term bioavailability of TNF and affecting the immune response [31].

The discussion emphasizes the need for a better understanding of the role of the TNF/TNFRI axis, especially in the post-infection context, as elevated sTNF-α levels in post-COVID individuals have been previously correlated with long COVID syndrome [32].

Under physiological conditions, the TNF-α/TNFRI complex does not harm endothelial cells, but excess TNF can activate apoptotic pathways and alter endothelial barrier permeability, contributing to a “pro-inflammatory” state [33]. Endothelial cell stress increases TNFRI expression and shedding, thereby contributing to soluble TNFRI levels [34]. In COVID-19, the coupling of sTNF-α/sTNFRI may lead to a prolonged release of sTNF-α, contributing to continuous and exacerbated inflammation, endothelial damage, and tissue injury.

This study emphasizes that sTNFRI acts as a sensitive indicator of immune response activation in COVID-19 patients. No other variable analyzed in the study demonstrated inferential power similar to sTNFRI. The sTNFRI/sTNFRII ratio, reflecting the biological effects of both receptors, also displayed significant inferential power. Binomial logistic regression modeling, combining the sTNFRI/sTNFRII ratio with CRP values, showed promising results in predicting clinical outcomes.

Given the multifactorial nature of the disease and the lack of consistent clinical management protocols during the survey period of the present study, there was difficulty in accessing metadata directly associated with patient deaths. This was one of the main limitations of the study.

While previous studies have identified cytokines like IL-6, IL-10, and TNF-α as early severity predictors or associated with mortality in severe COVID-19 [35,36], this study emphasizes the unique contribution of sTNFRI and sTNFRII levels. The joint use of these inflammatory mediators is proposed for assisting in the clinical management of hospitalized COVID-19 patients, potentially aiding in predicting the need for intensive treatment. The study suggests the development of low-cost tests for measuring sTNFRI and sTNFRII and mathematical models incorporating these parameters with routine CRP values.

## Figures and Tables

**Table 1:** Effect of blood cell variables among patients who were discharged from hospital or died related to severe COVID-19.

BLOOD CELLS							
Variables	Outcome	N	Ẋ//Md	σ	RBC—%	Power—%	*p*-Value
Erythrocytes—10^6^/mm^3^	Survivor	139	4.34//4.41	0.68	35.85	90.00	<0.001
	Deceased	142	3.88//3.81	0.85			
Hemoglobin—g%	Survivor	139	12.95//13.10	1.91	34.25	87.56	<0.001
	Deceased	142	11.63//11.80	2.41			
Hematocrit—%	Survivor	139	38.98//39.50	5.67	29.24	77.15	<0.001
	Deceased	142	54.45//35.55	223.78			
Platelets—10^3^/mm^3^	Survivor	139	226.00//205.00	89.3	17.15	40.36	0.821
	Deceased	142	212.00//205.00	83.6			
Leukocytes—mm^3^	Survivor	139	10,498.13//8800.00	5852.82	25.19	66.01	<0.001
	Deceased	142	13,000.22//11,735.00	6877.21			
Neutrophils—mm^3^	Survivor	139	8179.47//6966.00	4985.90	28.66	75.69	<0.001
	Deceased	141	10,748.47//9877.00	6038.81			
Band cell—mm^3^	Survivor	139	489.47//261.00	890.07	14.81	33.18	0.031
	Deceased	141	730.42//392.00	1036.80			
Lymphocytes—mm^3^	Survivor	139	1221.66//1014.00	679.17	32.16	83.70	<0.001
	Deceased	142	881.98//815.50	468.95			
Monocytes—mm^3^	Survivor	139	561.31//515.00	320.52	11.32	23.57	0.101
	Deceased	142	545.79//442.00	433.50			
Eosinophil—mm^3^	Survivor	138	25.21//0.00	55.43	0.54	5.47	0.915
	Deceased	141	35.23//0.00	85.32			
CRP—mg/dL	Survivor	87	83.10//58.70	69.00	42.43	83.04	<0.001
	Deceased	73	159.00//130.00	113.00			

N: sample number; Ẋ: mean; Md: median; σ: standard deviation; RBC: rank biserial correlation; %: percentage; CRP: C-reactive protein. The Mann–Whitney U test was used. Results were considered statistically significant at *p* < 0.05.

**Table 2:** Effect of immunological biomarkers among patients who were discharged from hospital or died related to severe COVID-19.

INFLAMMATORY MOLECULES							
Variable	Outcome	N	Ẋ//Md	σ	RBC—%	Power—%	*p*-Value
IL-12 p70—pg/mL	Survivor	153	4.58//4.05	2.51	2.58	7.78	0.690
	Deceased	168	4.62//4.10	2.42			
IL-1 β—pg/mL	Survivor	153	7.14//6.73	2.46	3.38	8.85	0.601
	Deceased	168	7.40//6.96	3.14			
IL-8—pg/mL	Survivor	153	178.58//21.95	988.86	13.9	33.28	0.032
	Deceased	168	239.11//36.11	1342.21			
IL-17—pg/mL	Survivor	153	54.67//26.50	142.13	7.72	16.57	0.232
	Deceased	168	65.91//18.87	134.45			
IFN-γ—pg/mL	Survivor	153	11.52//6.87	12.14	17.36	44.82	0.007
	Deceased	168	8.85//5.73	9.64			
IL-10—pg/mL	Survivor	153	14.88//9.18	24.97	20.25	54.85	0.002
	Deceased	168	30.62//15.15	65.37			
IL-6—pg/mL	Survivor	153	99.12//25.48	471.98	30.72	84.98	<0.001
	Deceased	168	377.98//73.59	1420.58			
IL-4—pg/mL	Survivor	153	14.62//10.76	10.96	19.83	53.40	0.002
	Deceased	168	11.32//8.86	7.58			
IL-2—pg/mL	Survivor	153	5.95//6.28	3.15	12.76	29.75	0.048
	Deceased	168	5.37//5.11	3.24			
Adiponectin—µg/mL	Survivor	147	5.38 × 10^6^//3.85 × 10^6^	3.74 × 10^6^	6.28	13.44	0.339
	Deceased	164	6.16 × 10^6^//4.95 × 10^6^	4.89 × 10^6^			
Leptin—ng/mL	Survivor	147	30,052.30//21,348.80	29,388.90	16.69	41.60	0.011
	Deceased	164	26,778.00//12,097.00	34,571.00			
MIF—pg/mL	Survivor	153	5299.00//3655.55	5342.10	22.12	61.22	<0.001
	Deceased	168	7549.00//4732.00	7484.00			
sCD40L—pg/mL	Survivor	151	1928.80//1952.70	979.20	19.53	52.03	0.003
	Deceased	167	1623.00//1539.00	1082.00			

N: sample number; Ẋ: mean; Md: median; σ: standard deviation; RBC: rank biserial correlation; %: percentage; IL: interleukin; CD: cluster differentiation; INF: interferon; MIF: macrophage migration inhibitory factor; s: soluble. The Mann–Whitney U test was used. Results were considered statistically significant at *p* < 0.05.

**Table 3:** Effect of TNF family of biomarkers among patients who were discharged from hospital or died related to severe COVID-19.

RECEPTOR FOR TNF AND THE RATIO							
Variable	Outcome	N	Ẋ//Md	σ	RBC or Cohen’s d—%	Power—%	*p*-Value
sTNF-α—pg/mL	Survivor	154	7.75//4.48	7.45	2.69	7.93	0.677
	Deceased	168	7.44//5.66	6.53			
sTNFRI—pg/mL	Survivor	154	2034.00//1508.00	1629.00	54.58	99.91	<0.001
	Deceased	168	5224.00//3260.00	5840.00			
sTNFRII—pg/mL	Survivor	154	5760.00//5997.00	1855.00	11.12	25.04	0.085
	Deceased	168	5356.00//5453.00	2100.00			
sTNFRI/sTNF-α	Survivor	154	496.00//388.00	528.00	35.80	94.03	<0.001
	Deceased	168	1141.00//590.00	1392.00			
sTNFRII/sTNF-α	Survivor	154	1555.00//1246.00	1245.00	9.31	20.84	0.149
	Deceased	168	1341.00//1014.00	1222.00			
sTNFRI/sTNFRII	Survivor	154	0.419//0.245	0.49	51.90	99.86	<0.001
	Deceased	168	1.33//0.590	1.70			

N: sample number; Ẋ: mean; Md: median; σ: standard deviation; RBC: rank biserial correlation; %: percentage; TNF: tumor necrosis factor; TNFRI: tumor necrosis factor receptor I; TNFRII: tumor necrosis factor receptor II; s: soluble. The Mann–Whitney U test was used. Results were considered statistically significant at *p* < 0.05.

**Table 4:** Binomial logistic regression model for predicting the prognosis of critically ill patients with COVID-19, using sTNFRI/sTNFRII ratio.

**Predictor (Parameter) ^1^**	**Odds Ratio**	**95% Confidence Interval**		***p*-Value**
		**Lower**	**Upper**	
Hemoglobin (g%)	0.7948	0.599	1.0600	0.112
Neutrophils (%)	1.0452	0.988	1.1100	0.122
CRP (mg/L)	1.0073	1.002	1.0100	0.011
sTNFRI/sTNFRII ratio	7.2332	1.857	28.1700	0.004
**Performance Metrics**				
Accuracy	0.740	Specificity	0.870	
AUC	0.830	Sensitivity	0.520	
**Predictor (Parameter) ^1^**	**Odds ratio**	**95% Confidence Interval**		***p*-Value**
		**Lower**	**Upper**	
CRP (mg/L)	1.0084	1.0032	1.0140	0.002
sTNFRI/sTNFRII ratio	8.3405	2.4201	28.7450	<0.001
**Performance Metrics**				
Accuracy	0.770	Specificity	0.900	
AUC	0.810	Sensitivity	0.590	
**Predictor (Parameter) ^1^**	**Odds ratio**	**95% Confidence Interval**		***p*-Value**
		**Lower**	**Upper**	
sTNFRI/sTNFRII ratio	3.9740	2.0700	7.6280	<0.001
**Performance Metrics**				
Accuracy	0.680	Specificity	0.920	
AUC	0.740	Sensitivity	0.370	

Legend: ^1^ Analyses refer to death/discharge; CRP: C-reactive protein; AUC: area under curve; %: percentage; TNFRI: tumor necrosis factor receptor I; TNFRII: tumor necrosis factor receptor II; s: soluble. Results were considered statistically significant at *p* < 0.05.

## Data Availability

The data presented in this study are available on request from the corresponding author due to ethical reasons.

## Supplementary Materials

The following supporting information can be downloaded at https://www.mdpi.com/article/10.3390/biomedicines12092138/s1, Table S1: Soluble Protein Measurement Details; Table S2: Participants’ General Characteristics; Table S3: Laboratory Findings—Red, White, and Platelet Series; Table S4: TNF Family Soluble Factors versus Sex; Table S5: TNF Family Soluble Factors versus Sex/Outcomes; Table S6: TNF Family Soluble Factors versus Age ≤ 59 and ≥60 years; Table S7: TNF Family Soluble Factors versus Sex/Outcomes; Table S8: Binomial Logistic Regression Model for Predicting the Prognosis of Patients with COVID-19, Using sTNFRI.
