# Supplementary material for: COVID-19 Inflammatory Syndrome: Lessons from TNFRI and CRP about the Risk of Death in Severe Disease
Source: Biomedicines. 2024 Sep 20;12(9):2138. doi: 10.3390/biomedicines12092138 (PMC11428742; doi:10.3390/biomedicines12092138)
Supplement: Supplementary file 1 [file biomedicines-12-02138-s001.zip › biomedicines-3128925-supplementary.pdf]

COVID inflammatory syndrome: lesson from TNFRI and CRP about the risk of death in severe disease

METHODS:

Procedures and Cytokine Measurement: The blood was drawn into tubes without anticoagulant, followed by centrifugation, and the resulting serum was stored at -80°C until it was ready for quantifying soluble factors. Soluble proteins were measured by Enzyme Linked-Immunosorbent Assay (ELISA) or Cytometric Bead Array (CBA), using commercial kits, according to the manufacturers' recommendations. The data were collected using an absorbance reader, EnSpire® (PerkinElmer, USA), or a flow cytometer, FACS Calibur, before being analyzed on FCAP software. In both cases, the concentrations of cytokines and soluble factors were determined based on a standard curve. Table S1 shows the targets and dilutions used in the samples to determine serum levels.

TABLES LEGENDS:

Table S1: Soluble Protein Measurement Details. Legend: Th: T helper cytokine profile; CD: cluster differentiation; IL: interleukin; IFN: interferon; TNF: tumor necrosis factor; TNFRI: tumor necrosis factor receptor I; TNFRII: tumor necrosis factor receptor II; s: soluble.

Table S2: Participants general characteristics. Legend: HD/COVID^{neg}: health donor without previous COVID-19 infection; AP/COVID^{pos}: Patients with active COVID-19; DP/COVID^{pos}: Patients with active COVID-19 who died and SP/COVID^{pos}: Patients with active COVID-19 who survived. n: sample number; %: percentage; y: years; d: days; σ = standard deviation; min: minimum value; max: maximum value; IC95%; confidence interval. [1] p-value between Health Donor versus Active Patients groups (HD/COVID^{neg} vs. AP/COVID^{pos}); [2] p value between Deceased Patients versus Survivor Patients groups (DP/COVID^{pos} vs. SP/COVID^{pos}); [3] p value between Female versus Male groups; [a] Fisher's exact test; [b] Ordinary one-way ANOVA or Kruskal Wallis, Dunn's post-test; [c] Unpaired t-test or Mann Whitney test. Results were considered statistically significant at $p<0.05$.

Table S3: Laboratory Findings – Red, White and Platelet Series. Legend: HD/COVID^{neg}: health donor without previous COVID-19 infection; AP/COVID^{pos}: Patients with active COVID-19; DP/COVID^{pos}: Patients with active COVID-19 who deceased and SP/COVID^{pos}: Patients with active COVID-19 who survived. RBC: Reed Blood Cell; Hb: Hemoglobin; Hct: Hematocrit; WBC: White Blood Cell; PMN: Polymorphonuclear; Min: minimum; Max: maximum; IC95%; confidence interval. p-value[1]: between Deceased Patients versus Survivor Patients groups (DP/COVID^{pos} vs. SP/COVID^{pos}); p-value[2]: between Female versus Male groups; [a]Unpaired t test or Mann Whitney test; [b]Ordinary one-way ANOVA or Kruskall Wallis, Dunn's post test; Results were considered statistically significant at $p < 0.05$.

Table S4: TNF Family Soluble Factors versus Sex. Legend: TNF: tumor necrosis factor; TNFRI: tumor necrosis factor receptor I; TNFRII: tumor necrosis factor receptor II; s: soluble; %: percentage; Std: standard. Statistical tests used are indicated in the tableResults were considered statistically significant at $p<0.05$.

Table S5: TNF Family Soluble Factors versus Sex/Outcomes. Legend: TNF: tumor necrosis factor; TNFRI: tumor necrosis factor receptor I; TNFRII: tumor necrosis factor receptor II; s: soluble; %: percentage; Std: standard. Statistical tests used are indicated in the table. Results were considered statistically significant at $p<0.05$.

Table S6: TNF Family soluble factors versus Age ≤59 and ≥60 years. Legend: TNF: tumor necrosis factor; TNFRI: tumor necrosis factor receptor I; TNFRII: tumor necrosis factor receptor II; s: soluble; %: percentage; Std: standard. Statistical tests used are indicated in the table. Results were considered statistically significant at $p<0.05$.

Table S7: TNF Family Soluble Factors versus Sex/Outcomes. Legend: TNF: tumor necrosis factor; TNFRI: tumor necrosis factor receptor I; TNFRII: tumor necrosis factor receptor II; s: soluble; %: percentage; Std: standard. Statistical tests used are indicated in the table. Results were considered statistically significant at $p<0.05$.

Table S8: Binomial logistic regression model for predicting the prognosis of patients with COVID-19, using sTNFRI. Legend: [1]analyses refer to death/discharge; CRP: C-reactive protein; AUC: area under curve; % percentage; TNFRI: tumor necrosis factor receptor I; TNFRII: tumor necrosis factor receptor II; s: soluble. Results were considered statistically significant at $p<0.05$.

Table S1 - Soluble Protein Measurement Details

Kit Name	Manufacture	Cat #	Targets	Dilution Sample
Human Th1/Th2/Th17 Cytokine Kit	BD Bioscience	560484	IL-2; IL-4; IL-6; IL-10; TNF; IFNγ; IL-17A	-
Human Inflammatory Cytokines Kit	BD Bioscience	551811	IL-8; IL-1β; IL12p70	-
Human TNF RI/TNFRSF1A Duoset ELISA	R&D Systems	DY225	sTNFRI	1:2
Human sTNF RII/TNFRSF1B Duoset ELISA	R&D Systems	DY726	sTNFRII	1:2
Human CD40L/TNFSF5 Duoset ELISA	R&D Systems	DY617	sCD40L	1:2
Human MIF Duoset ELISA	R&D Systems	DY289	sMIF	1:10
Human Adiponectin/Acrp30 Duoset ELISA	R&D Systems	DY1065	sAdiponectin	1:2000
Human Leptin Duoset ELISA	R&D Systems	DY398	sLeptin	1:50

Table S2 – Participants general characteristics

	HD/COVID-19^{NEG}	AP/COVID-19^{POS}	DP/COVID-19^{POS}	SP/COVID-19^{POS}
**N (%)**	14	214 (100)	90 (42.06)	124 (57.94)

SEX

	HD N	HD %	AP N	AP %	*p*-Value [1]	DP N	DP %	SP N	SP %	*p*-Value[2]
**Female**	9	64.29	71	33.18		29	32.22	42	33.87	
**Male**	5	35.71	143	66.82	0.0387[a]	61	67.78	82	66.13	0.8833[a]
**Total**	14	100.0	214	100.0		90	100.0	124	100.0	

AGE

	HD Mean (min - max), y	HD σ	HD *p*-Value [3]	AP Mean (min - max), y	AP σ	AP *p*-Value [3]	*p*-Value [1]	DP Mean (min - max), y	DP σ	DP *p*-Value [3]	SP Mean (min - max), y	SP σ	SP *p*-Value [3]	*p*-Value [2]
	46.36 (22.0 - 80.0)	19.15		58.42 (14.0 - 98.0)	16.07		0.0156 [b]	65.14 (23.0 - 98.0)	16.00		53.44 (14.0 - 92.0)	14.21		<0.0001[b]
**Female**	41.22 (22.0 - 77.0)	17.80	0.1885 [c]	57.25 (14.0 - 98.0)	19.55	0.662 [c]	0.2659 [b]	64.86 (23.0 - 98.0)	18.46	0.9299 [c]	52.0 (14.0 - 92.0)	18.72	0.4208 [c]	0.0414 [b]
**Male**	55.6 (25.0 - 80.0)	19.78		59.0 (29.0 - 89.0)	14.06		>0.9999 [b]	65.39 (36.0 - 89.0)	14,92		54.19 (29.0 - 88.0)	11.25		0.0006 [b]

TIME FROM FIRST SYMPTOM TO SAMPLING

	AP Median (min - max), d	AP IC95%	AP *p*-Value [3]	DP Median (min - max), d	DP IC95%	DP *p*-Value [3]	SP Median (min - max), d	SP IC95%	SP *p*-Value [3]	*p*-Value [2]
	9.0 (1.0 - 48.0)	95.28		8.0 (1.0 - 30.0)	95.54		9.0 (1.0 - 48.0)	96.16		0.5810 [b]
**Female**	8.0 (1.0 - 48.0)	96.81	0.6366 [c]	8.0 (1.0 - 30.0)	97.59	0.9090 [c]	9.0 (1.0 - 48.0)	95.64	0.4385 [c]	>0.9999 [b]
**Male**	9.0 (2.0 - 25.0)	95.56		8.0 (2.0 - 23.0)	96.04		9.0 (2.0 - 25.0)	96.48		>0.9999 [b]

TIME FROM FIRST SYMPTOM TO OUTCOME

	AP Median (min - max), d	AP IC95%	AP p-value[3]	DP Median (min - max), d	DP IC95%	DP p-value[3]	SP Median (min - max), d	SP IC95%	SP p-value[3]	p-value[2]
	16.0 (1.0 - 70.0)	96.04		19.0 (4.0 - 58.0)	96.66		15.0 (1.0 - 70.0)	96.16		0.1207 [b]
**Female**	15.0 (1.0 - 63.0)	96.81	0.3630 [c]	16.0 (5.0 - 34.0)	97.59	0.2169 [c]	14.5 (1.0 - 63.0)	95.64	0.8548 [c]	>0.9999 [b]
**Male**	17.0 (4.0 - 70.0)	95.56		19.0 (4.0 - 58.0)	96.04		15.0 (4.0 - 70.0)	96.48		0.3664 [b]

Table S3: Laboratory Findings – Red, White and Platelet Series

	AP/COVID-19^POS			DP/COVID-19^POS			SP/COVID-19^POS			
					**RBC x 10^6/mm^3**					
	**Median (min - max)**	**IC95%**	***p*-Value [2]**	**Median (min - max)**	**IC95%**	***p*-Value [2]**	**Median (min - max)**	**IC95%**	***p*-Value [2]**	***p*-Value [1]**
	4.31 (2.06 - 6.09)	96.14		4.21 (2.06 - 6.02)	95.54		4.44 (2.62 - 6.09)	95.49		0.0048 [a]
**Female**	4.03 (2.41 - 5.29)	97.05	<0.0001[a]	4.03 (2.41 - 5.19)	97.59	0.2965 [a]	4.12 (2.62 - 5.29)	96.15	<0.0001[a]	0.9996 [b]
**Male**	4.475 (2.06 - 6.09)	96.45		4.27 (2.06 - 6.02)	96.04		4.65 (3.06 - 6.09)	95.52		0.0047 [b]
					**Hb (g/dL)**					
	**Median (min - max)**	**IC95%**	***p*-Value [2]**	**Median (min - max)**	**IC95%**	***p*-Value [2]**	**Median (min - max)**	**IC95%**	***p*-Value [2]**	***p*-Value [1]**
	13.0 (6.20 - 17.10)	96.14		12.40 (6.20 - 16.60)	95.54		13.20 (7.70 - 17.10)	95.49		0.0053 [a]
**Female**	12.2 (7.30 - 15.10)	97.05	<0.0001[a]	12.10 (7.30 - 14.80)	97.59	0.0524 [a]	12.25 (7.70 - 15.10)	96.15	<0.0001[a]	>0.9999 [b]
**Male**	13.45 (6.20 - 17.10)	96.45		12.80 (6.20 - 16.60)	96.04		13.70 (9.70 - 17.10)	95.52		0.0534 [b]
					**Hct (%)**					
	**Median (min - max)**	**IC95%**	***p*-Value [2]**	**Median (min - max)**	**IC95%**	***p*-Value [2]**	**Median (min - max)**	**IC95%**	***p*-Value [2]**	***p*-Value [1]**
	39.10 (20.0 - 51.10)	96.14		38.20 (20.0 - 50.50)	95.54		39.70 (23.40 - 51.10)	95.49		0.0183 [a]
**Female**	37.0 (22.20 - 46.50)	97.05	<0.0001[a]	37.40 (22.20 - 45.90)	97.59	0.1708 [a]	36.80 (23.40 - 46.50)	96.15	<0.0001 [a]	0.9879 [b]
**Male**	40.25 (20.0 - 51.10)	96.45		39.20 (20.0 - 50.50)	96.04		41.60 (30.60 - 51.10)	95.52		0.0107 [b]
					**WBC/mm^3**					
	**Median (min - max)**	**IC95%**	***p*-Value [2]**	**Median (min - max)**	**IC95%**	***p*-Value [2]**	**Median (min - max)**	**IC95%**	***p*-Value [2]**	***p*-Value [1]**
	9,100 (2,400 – 44,800)	96.14		9,900 (2,480 – 27,800)	95.54		8,400 (2,700 – 44,800)	95.49		0.0556 [a]
**Female**	8,750 (2,700 – 24,240)	97.05	0.1110 [a]	9,900 (4,000 – 22,260)	97.59	0.5264 [a]	7,950 (2,700 – 24,240)	96.16	0.1075 [a]	>0.9999 [b]
**Male**	9,210 (2,480 – 44,800)	96.45		10,280 (2,480 – 27,800)	96.04		8,800 (2,800 – 44,800)	95.52		>0.9999 [b]
					**PMN/mm^3**					
	**Median (min - max)**	**IC95%**	***p*-Value [2]**	**Median (min - max)**	**IC95%**	***p*-Value [2]**	**Median (min - max)**	**IC95%**	***p*-Value [2]**	***p*-Value [1]**
	7,680 (1,042 – 43,456)	96.14		8,644 (1,042 – 26,966)	95.54		6,885 (1,512 – 43,456)	95.49		0.0093 [a]
**Female**	7,052 (1,620 – 21,574)	97.05	0.0726 [a]	8,428 (3,320 – 18,921)	97.59	0.4007 [a]	6,297 (1,620 – 21,574)	96.15	0.0911 [a]	0.9964 [b]
**Male**	7,838 (1,042 – 43,414)	96.45		9,252 (1,042 – 26,966)	96.04		7,140 (1,512 – 43,456)	95.52		0.9285 [b]
					**Lymphocytes/mm^3**					
	**Median (min - max)**	**IC95%**	***p*-Value [2]**	**Median (min - max)**	**IC95%**	***p*-Value [2]**	**Median (min - max)**	**IC95%**	**p-value[2]**	***p*-Value [1]**
	913.0 (0.0 – 4,794)	96.14		808.0 (0.0 – 2,671)	95.54		1,014 (4.0 – 4,794)	95.49		<0.0001 [a]
**Female**	1,065 (168.0 – 3,045)	97.05	0.0243 [a]	940.0 (168.0 – 2,671)	97.59	0.1399 [a]	1,208 (386.0 – 3,045)	96.15	0.0921 [a]	0.2177 [b]
**Male**	844.0 (0.0 – 4,794)	96.45		780.0 (0.0 – 1,548)	96.04		924.0 (4.0 – 4,794)	95.52		0.0056 [b]
					**Monocytes/mm^3**					
	**Median (min - max)**	**IC95%**	***p*-Value [2]**	**Median (min - max)**	**IC95%**	***p*-Value [2]**	**Median (min - max)**	**IC95%**	**p-value[2]**	***p*-Value [1]**
	474.0 (0.0 – 1,998)	96.14		413.0 (0.0 – 1,485)	95.54		510.0 (2.0 – 1,998)	95.49		0.0071 [a]
**Female**	476.0 (129.0 – 1,317)	97.05	0.9079 [a]	475.0 (129.0 – 1,252)	97.59	0.5015 [a]	486.0 (140.0 – 1,317)	96.15	0.4661 [a]	>0.9999 [b]
**Male**	464.0 (0.0 – 1,998)	96.45		364.0 (0.0 – 1,485)	96.04		515.0 (2.0 – 1,998)	95.52		0.0878 [b]
					**Platelets x 10^3/mm^3**					
	**Median (min - max)**	**IC95%**	***p*-Value [2]**	**Median (min - max)**	**IC95%**	***p*-Value [2]**	**Median (min - max)**	**IC95%**	**p-value[2]**	***p*-Value [1]**
	201.0 (82.0 - 752.0)	96.14		204.5 (82.0 - 401.0)	95.54		198.0 (108.0 - 752.0)	95.49		0.4873 [a]
**Female**	210.0 (82.0 - 532.0)	97.05	0.5661 [a]	189.0 (82.0 - 401.0)	97.59	0.8784 [a]	223.0 (108.0 - 532.0)	96.15	0.4348 [a]	>0.9999 [b]
**Male**	197.0 (86.0 - 752.0)	96.45		209.0 (86.0 - 373.0)	96.04		188.0 (119.0 - 752.0)	95.52		>0.9999 [b]

Table S4: TNF Family Soluble Factors versus Sex

SOLUBLE FACTORS versus SEX								
	**sTNFα**		**sTNFRI**		**sTNFRII**		**sCD40L**	
	**Men**	**Women**	**Men**	**Women**	**Men**	**Women**	**Men**	**Women**
**Number of values**	143	71	143	71	143	71	140	70
**n total**	214		214		214		210	
**Minimum**	1.06	1.59	256.1	474.5	0	833.6	0	178.2
**25% Percentile**	2.48	2.65	1,281	1,181	4,995	4,712	1,134	1,163
**Median**	3.72	5.43	1,906	1,728	6,045	6,002	1,863	1,866
**75% Percentile**	8.36	9.76	3,294	2,983	7,243	7,033	2,549	2,639
**Maximum**	28.1	34.48	21,000	11,546	8,608	9,791	5,494	7,016
**Range**	27.04	32.89	20,744	11,071	8,608	8,957	5,494	6,838
**95% CI of median**								
**Actual confidence level**	95.56%	96.81%	95.56%	96.81%	95.56%	96.81%	96.58%	95.86%
**Lower confidence limit**	3.07	3.41	1,747	1,451	5,698	5,383	1,592	1,474
**Upper confidence limit**	4.64	7.88	2,173	2,114	6,451	6,494	2,019	2,235
**Mean**	6.711	8.162	2,974	2,363	5,833	5,736	1,841	1,928
**Std. Deviation**	6.768	7.609	3,060	1,995	1,853	1,972	1,051	1,075
**Std. Error of Mean**	0.5659	0.903	255.9	236.8	155	234	88.82	128.5
**Lower 95% CI of mean**	5.592	6.361	2,468	1,891	5,526	5,269	1,665	1,672
**Upper 95% CI of mean**	7.83	9.963	3,479	2,836	6,139	6,203	2,016	2,185
	**Mann-Whitney test**		**Mann-Whitney test**		**Mann-Whitney test**		**Unpaired t test**	
	p-Value	0.0973	*p*-Value	0.2161	*p*-Value	0.7226	*p*-Value	0.5726

Table S5: TNF Family Soluble Factors versus Sex/Outcomes

SOLUBLE FACTORS versus SEX/OUTCOMES								
	sTNFα				sTNFRI			
	Survivor Men	Deceased Men	Survivor Women	Deceased Women	Survivor Men	Deceased Men	Survivor Women	Deceased Women
**Number of values**	82	61	42	29	82	61	42	29
**n total**	214				214			
**Minimum**	1.06	1.3	1.59	1.91	256.1	789.3	474.5	474.5
**25% Percentile**	2.463	2.495	2.643	2.82	1,156	1845	995.5	1,634
**Median**	4.165	3.32	4.35	6.93	1,508	2,704	1,340	2,312
**75% Percentile**	8.613	7.47	9.86	9.31	2,589	5,145	2,079	4,015
**Maximum**	28.1	27.85	34.48	24.31	13,170	21,000	4,530	11,546
**Range**	27.04	26.55	32.89	22.4	12,914	20,211	4,056	11,071
**95% CI of median**								
**Actual confidence level**	96.48%	96.04%	95.64%	97.59%	96.48%	96.04%	95.64%	97.59%
**Lower confidence limit**	3.2	2.84	3.03	3.37	1,310	2,094	1,156	1,691
**Upper confidence limit**	5.48	5.27	8.2	8.04	1,847	3,662	1,787	3,860
**Mean**	7.013	6.305	8.593	7.538	2,084	4,169	1,692	3,336
**Std. Deviation**	6.943	6.56	8.758	5.634	1,885	3,852	982.6	2,623
**Std. Error of Mean**	0.7667	0.8399	1.351	1.046	208.1	493.2	151.6	487.1
**Lower 95% CI of mean**	5.488	4.625	5.864	5.395	1,670	3,183	1,385	2,338
**Upper 95% CI of mean**	8.539	7.985	11.32	9.681	2,498	5,156	1,998	4,334
	**Mann-Whitney test**		**Mann-Whitney test**		**Mann-Whitney test**		**Mann-Whitney test**	
	p-Value	0.5248	*p*-Value	0.7077	*p*-Value	<0.0001	*p*-Value	<0.0001

SOLUBLE FACTORS versus SEX/OUTCOMES (continue)								
	**sTNFRII**				**sCD40L**			
	**Survivor Men**	**Deceased Men**	**Survivor Women**	**Deceased Women**	**Survivor Men**	**Deceased Men**	**Survivor Women**	**Deceased Women**
**Number of values**	82	61	42	29	80	60	41	29
**n total**	214				210			
**Minimum**	0	1,411	833.6	1,359	0	0	187	178.2
**25% Percentile**	5,235	4,428	4,863	4,661	1,251	934.5	1,339	901
**Median**	6,216	5,572	6,129	5,783	1,969	1,691	1,966	1,785
**75% Percentile**	7,148	7,481	7,404	6,851	2,542	2,606	2,768	2,551
**Maximum**	8,342	8,608	9,791	8,388	5,494	4,664	3,553	7,016
**Range**	8,342	7,198	8,957	7,029	5,494	4,664	3,366	6,838
**95% CI of median**								
**Actual confidence level**	96.48%	96.04%	95.64%	97.59%	96.70%	97.27%	97.25%	97.59%
**Lower confidence limit**	5,827	5,208	5,289	4,938	1,608	1,290	1,474	946.8
**Upper confidence limit**	6,625	6,752	6,610	6,741	2,178	2,025	2,259	2,462
**Mean**	5,991	5,621	5,890	5,513	1,938	1,711	1,943	1,908
**Std. Deviation**	1,652	2,089	2,022	1,909	1,063	1,029	864.3	1,334
**Std. Error of Mean**	182.4	267.5	312.1	354.5	118.9	132.8	135	247.8
**Lower 95% CI of mean**	5,628	5,085	5,260	4,787	1,702	1,445	1,670	1,400
**Upper 95% CI of mean**	6,353	6,156	6,520	6,240	2,175	1,977	2,216	2,415
	**Mann-Whitney test**		**Unpaired t test**		**Unpaired t test**		**Unpaired t test**	
	p-Value	0.3698	*p*-Value	0.4331	*p*-Value	0.2062	*p*-Value	0.8925

Table S6 – TNF Family soluble factors versus Age ≤59 and ≥60 years

SOLUBLE FACTORS versus Age≤59 and ≥60years								
	**sTNFα**		**sTNFRI**		**sTNFRII**		**sCD40L**	
	**≤59 years**	**≥60 years**	**≤59 years**	**≥60 years**	**≤59 years**	**≥60 years**	**≤59 years**	**≥60 years**
**Number of values**	118	95	118	95	118	95	116	93
**n total**	213		213		213		209	
**Minimum**	1.14	1.06	256.1	843.5	0	0	0	0
**25% Percentile**	2.48	2.65	1,084	1,706	4,938	4,712	1,251	951.5
**Median**	3.63	4.6	1,568	2,728	6,047	5,992	2,009	1,624
**75% Percentile**	8.04	10.05	2,228	4,205	7,194	7,044	2,689	2,271
**Maximum**	28.1	34.48	12,721	21,000	9,791	8,670	4,664	7,016
**Range**	26.96	33.42	12,465	20,157	9,791	8,670	4,664	7,016
**95% CI of median**								
**Actual confidence level**	96.62%	96.04%	96.62%	96.04%	96.62%	96.04%	96.77%	96.25%
**Lower confidence limit**	2.92	3.45	1,310	2,094	5,766	5,482	1,800	1,349
**Upper confidence limit**	4.76	7.36	1,763	3,312	6,451	6,494	2,216	1,910
**Mean**	6.393	8.18	2,011	3,728	5,870	5,690	1,952	1,764
**Std. Deviation**	6.322	7.863	1,872	3,358	1,816	1,977	1,002	1,124
**Std. Error of Mean**	0.582	0.8068	172.3	344.5	167.2	202.9	93.06	116.5
**Lower 95% CI of mean**	5.241	6.579	1,670	3,044	5,539	5,288	1,767	1,533
**Upper 95% CI of mean**	7.546	9.782	2,353	4,412	6,201	6,093	2,136	1,996
	**Mann-Whitney test**		**Mann-Whitney test**		**Mann-Whitney test**		**Unpaired t test**	
	p-Value	0.1105	*p*-Value	<0.0001	*p*-Value	0.6735	*p*-Value	0.2041

Table S7: TNF Family Soluble Factors versus Sex/Outcomes

SOLUBLE FACTORS versus Outcomes/Age								
	sTNFα				sTNFRI			
	Survivor ≤59	Deceased ≤59	Survivor ≥60	Deceased ≥60	Survivor ≤59	Deceased ≤59	Survivor ≥60	Deceased ≥60
**Number of values**	85	33	38	57	85	33	38	57
**n total**	213				213			
**Minimum**	1.14	2	1.06	1.3	256.1	474.5	843.5	1,054
**25% Percentile**	2.445	2.51	3.033	2.55	1,042	1,586	1,294	2,081
**Median**	3.63	2.99	7.11	3.96	1,321	1,842	1,805	3,563
**75% Percentile**	8.2	7.775	11.65	8.765	2,021	2,667	3,016	5,578
**Maximum**	28.1	24.31	34.48	27.85	9,431	12,721	13,170	21,000
**Range**	26.96	22.31	33.42	26.55	9,175	12,247	12,327	19,946
**95% CI of median**								
**Actual confidence level**	97.05%	96.49%	96.64%	96.69%	97.05%	96.49%	96.64%	96.69%
**Lower confidence limit**	2.88	2.8	3.41	3.28	1,181	1,669	1,398	2,670
**Upper confidence limit**	4.7	6.27	9.3	7.36	1,611	2,312	2,709	4,205
**Mean**	6.626	5.795	9.61	7.227	1,752	2,680	2,408	4,607
**Std. Deviation**	6.826	4.824	8.96	6.96	1,396	2,657	2,057	3,763
**Std. Error of Mean**	0.7404	0.8398	1.453	0.9219	151.4	462.6	333.7	498.4
**Lower 95% CI of mean**	5.153	4.084	6.665	5.381	1,451	1,738	1,732	3,609
**Upper 95% CI of mean**	8.098	7.505	12.55	9.074	2,053	3,623	3,084	5,606
	**Mann-Whitney test**		**Mann-Whitney test**		**Mann-Whitney test**		**Mann-Whitney test**	
	p-Value	0.9251	*p*-Value	0.2395	*p*-Value	0.0062	*p*-Value	<0.0001

SOLUBLE FACTORS versus Outcomes/Age (continue)								
	sTNFRII				sCD40L			
	Survivor ≤59	Deceased ≤59	Survivor ≥60	Deceased ≥60	Survivor ≤59	Deceased ≤59	Survivor ≥60	Deceased ≥60
**Number of values**	85	33	38	57	83	33	37	56
**n total**	213				209			
**Minimum**	0	1,492	0	1,359	0	260.3	386.4	0
**25% Percentile**	5,010	4,609	5,237	4,122	1,222	1,389	1,371	774.5
**Median**	6,189	5,905	6,129	5,564	1,983	2,056	1,951	1,391
**75% Percentile**	7,084	7,734	7,304	6,915	2,649	2,743	2,389	2,243
**Maximum**	9,791	8,373	8,670	8,608	4,540	4,664	5,494	7,016
**Range**	9,791	6,881	8,670	7,250	4,540	4,404	5,108	7,016
**95% CI of median**								
**Actual confidence level**	97.05%	96.49%	96.64%	96.69%	95.25%	96.49%	95.30%	95.60%
**Lower confidence limit**	5,766	4,938	5,708	5,208	1,563	1,701	1,608	1,033
**Upper confidence limit**	6,509	7,170	6,799	6,495	2,216	2,551	2,202	1,750
**Mean**	5,889	5,823	6,053	5,449	1,898	2,088	2,027	1,591
**Std. Deviation**	1,761	1,979	1,825	2,053	1,011	982.2	984.2	1,184
**Std. Error of Mean**	191	344.6	296.1	271.9	111	171	161.8	158.2
**Lower 95% CI of mean**	5,509	5,121	5,453	4,904	1,677	1,739	1,699	1,274
**Upper 95% CI of mean**	6,269	6,525	6,653	5,993	2,118	2,436	2,355	1,908
	**Mann-Whitney test**		**Mann-Whitney test**		**Unpaired t test**		**Mann-Whitney test**	
	p-Value	0.9394	*p*-Value	0.1646	*p*-Value	0.3593	*p*-Value	0.0229

Table S8: Binomial logistic regression model for predicting the prognosis of patients with COVID-19, using sTNFRI

Predictor (Parameter)[1]	Odds ratio	95% Confidence Interval		p-Value
		Lower	Upper	
Hemoglobin (g%)	0.8670	0.7241	1.0400	0.121
Neutrophils (%)	1.0001	0.9999	1.0002	0.745
CRP (mg/L)	1.0080	1.0036	1.0100	<.001
sTNFRI (pg/mL)	1.0003	1.0002	1.0004	<.001
**Performance Metrics**				
Accuracy	0.725	Specificity	0.828	
AUC	0.824	Sensitivity	0.603	
**Predictor (Parameter)[1]**	**Odds ratio**	**95% Confidence Interval**		**p-Value**
		**Lower**	**Upper**	
CRP (mg/L)	1.0078	1.0032	1.0120	< .001
sTNFRI (pg/mL)	1.0005	1.0003	1.0010	< .001
**Performance Metrics**				
Accuracy	0.725	Specificity	0.828	
AUC	0.822	Sensitivity	0.603	
**Predictor (Parameter)[1]**	**Odds ratio**	**95% Confidence Interval**		**p-Value**
		**Lower**	**Upper**	
sTNFRI (pg/mL)	1.0009	1.0008	1.0010	< .001
**Performance Metrics**				
Accuracy	0.671	Specificity	0.766	
AUC	0.773	Sensitivity	0.583	
